# Concurrent Congenital Umbilicobiliary Fistula and Vesicourachal Diverticula in a Dog

**DOI:** 10.3390/ani15243626

**Published:** 2025-12-17

**Authors:** Sathidpak N. Assawarachan, Veerada Wachirodom, Benjang Hakhen, Piyathip Chuchalermporn, Rungrueang Yodsheewan, Phudit Maneesaay, John M. Cullen, Jonathan A. Lidbury, Panpicha Sattasathuchana

**Affiliations:** 1 Department of Companion Animal Clinical Sciences, Faculty of Veterinary Medicine, Kasetsart University, 50 Phaholyothin Rd., Lat Yao, Jatujak, Bangkok 10900, Thailand; sathidpak.n@ku.ac.th; 2 Endocrinology and Gastroenterology Unit, Kasetsart University Veterinary Teaching Hospital, Faculty of Veterinary Medicine, Kasetsart University, 50 Phaholyothin Rd., Lat Yao, Jatujak, Bangkok 10900, Thailand; veerada.w@ku.th; 3 Surgical Unit, Kasetsart University Veterinary Teaching Hospital, Faculty of Veterinary Medicine, Kasetsart University, 50 Phaholyothin Rd., Lat Yao, Jatujak, Bangkok 10900, Thailand; benjang.h@ku.th; 4 Radiology Unit, Kasetsart University Veterinary Teaching Hospital, Faculty of Veterinary Medicine, Kasetsart University, 50 Phaholyothin Rd., Lat Yao, Jatujak, Bangkok 10900, Thailand; piyathip.c@ku.th; 5 Department of Pathology, Faculty of Veterinary Medicine, Kasetsart University, 50 Phaholyothin Rd., Lat Yao, Jatujak, Bangkok 10900, Thailand; fvetrry@ku.ac.th (R.Y.); fvetpdm@ku.ac.th (P.M.); 6 College of Veterinary Medicine, North Carolina State University, Raleigh, NC 27607, USA; jcullen@ncsu.edu; 7 Gastrointestinal Laboratory, Department of Small Animal Clinical Sciences, School of Veterinary Medicine and Biomedical Sciences, Texas A&M University, College Station, TX 77843, USA; jlidbury@cvm.tamu.edu

**Keywords:** dogs, congenital abnormalities, biliary diseases, gallbladder agenesis, urachal anomalies

## Abstract

Congenital umbilicobiliary fistula is a rare disease reported in humans and dogs. A 2-month-old, intact, male French Bulldog presented with a greenish-yellow discharge dripping from the umbilicus since birth. Complete blood count and serum biochemistry were within normal limits, but serum alkaline phosphatase activity was mildly elevated. A positive contrast cystogram was performed to rule out a patent urachus and confirmed the presence of a vesicourachal diverticula, a type of urachal anomalies. An abdominal ultrasound and computed tomography fistulogram demonstrated a communication between the umbilicus and common bile duct, which suggested an umbilicobiliary fistula. Surgical correction of the umbilicobiliary fistula and vesicourachal diverticula was successfully performed with an uneventful recovery. Histological analysis of the fistulous tract demonstrated a cuboidal/columnar lining epithelium that transitioned to squamous epithelium near the umbilicus. Agenesis of the gallbladder was noted. The application of multiple imaging techniques in the diagnosis and surgical correction of these congenital abnormalities (umbilicobiliary fistula, gallbladder agenesis, and vesicourachal diverticula) was beneficial for treatment planning and outcome.

## Simple Summary

Umbilicobiliary fistula in dogs is a rare congenital malformation that has been rarely reported. Here, we demonstrated the first case report of concurrent congenital umbilicobiliary fistula, gallbladder agenesis, and vesicourachal diverticula in a two-month-old, intact, male French Bulldog. Umbilicobiliary fistula and vesicourachal diverticula were diagnosed by positive contrast cystography, abdominal ultrasonography, computed tomography fistulography, and histopathology. Successful surgical correction was achieved.

## 1. Introduction

An umbilicobiliary fistula is an extremely rare congenital hepatobiliary anomaly in humans and dogs [1,2,3,4]. It is an abnormal connection between the umbilicus and the biliary system during embryonic development, allowing for bile to drain through the umbilicus. The liver originates from the foregut endoderm, forming the hepatic diverticulum, which gives rise to two developmental buds. The cranial bud develops into the liver and the hilar biliary tract. The caudal bud differentiates into two portions: the superior portion forms the gallbladder and cystic duct, while the inferior portion gives rise to the ventral pancreas [5]. The excessive ventral evagination of the gallbladder or bile duct or thinning of the adjacent ventral body wall mesenchyme during development may permit an abnormal connection between the biliary system and the umbilicus. Previous reports in humans have shown aberrations of biliary embryogenesis involving the extra-hepatic biliary system opening into the respiratory and gastrointestinal tract [6,7].

Congenital disorders involving the urachus are uncommon in both dogs and cats. In normal development, the urachus—a fetal tubular connection linking the urinary bladder to the allantoic sac—regresses and closes completely at pre-birth [8,9]. The failure of the urachus to close partially or completely results in urachal anomalies. The exact cause of urachal remnants has not been identified; however, this anomaly occurs during fetal development.

To the authors’ knowledge, three cases of umbilicobiliary fistula have previously been reported in dogs within two decades [1,3,4]. The objective of this report is to describe the first clinical case in a dog presenting with three concurrent congenital abnormalities: an umbilicobiliary fistula, gallbladder agenesis, and a vesicourachal diverticula.

## 2. Case Description

**Figure 1:**
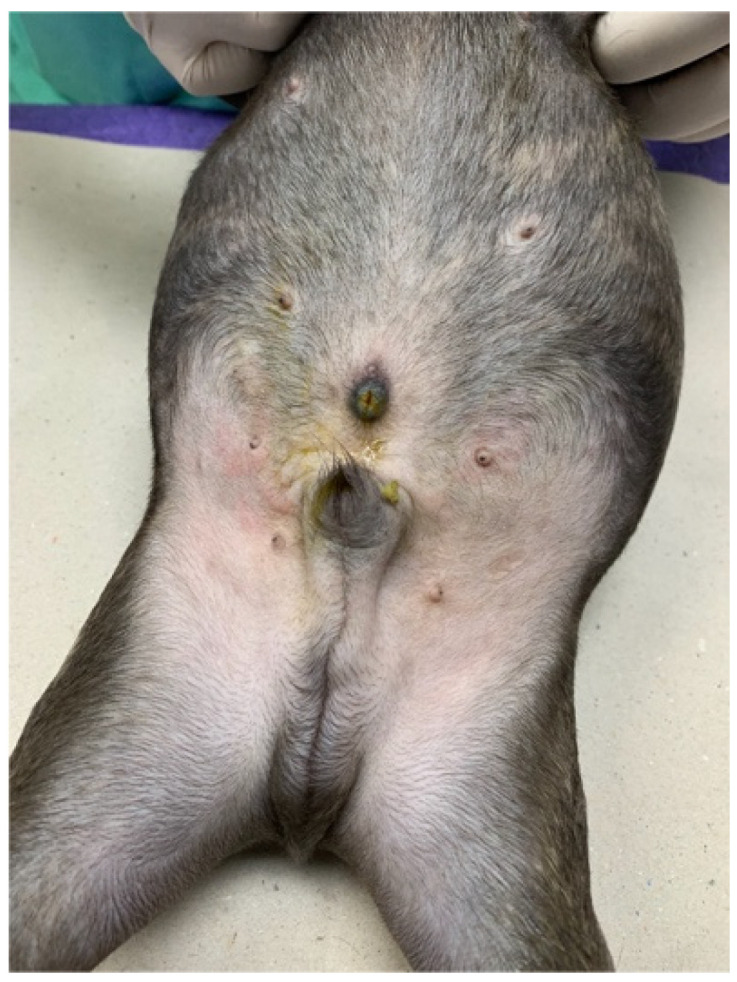
An open umbilicus was noted with bile-stained skin surrounding the umbilicus.

A 2-month-old, intact, male, French Bulldog (body weight 2.5 kg) was referred to Kasetsart University Veterinary Teaching Hospital for further investigation due to a history of greenish-yellow discharge draining from the umbilicus since birth (Figure 1). The puppy was alert and had a normal appetite. The puppy was fed commercially prepared, complete, and balanced puppy food. A complete physical examination was performed. There was no sign of icterus, and all vital parameters were within normal limits. An abdominal examination revealed no pain and no palpable mass. The periumbilical skin appeared normal on examination, but the umbilical orifice remained open.

Hematology and blood chemistry testing were performed using an automated hematology analyzer (Sysmex XN-1000TM Hematology Analyzer, Sysmex, IL, USA) and an automatic chemistry analyzer (IL Lab 650 chemistry system, Diamond Diagnostics, MA, USA), respectively. A complete blood count, serum creatinine, blood urea nitrogen, alanine aminotransferase (ALT), total bilirubin, total protein, albumin, and glucose concentrations were within normal limits. Serum alkaline phosphatase (ALP) concentration was mildly increased (165 U/L; reference interval [RI] 8–76 U/L). The laboratory results showed a urine specific gravity of 1.045 and a pH of 6.5, with no evidence of leukocyturia, hematuria, or other abnormalities.

**Figure 2:**
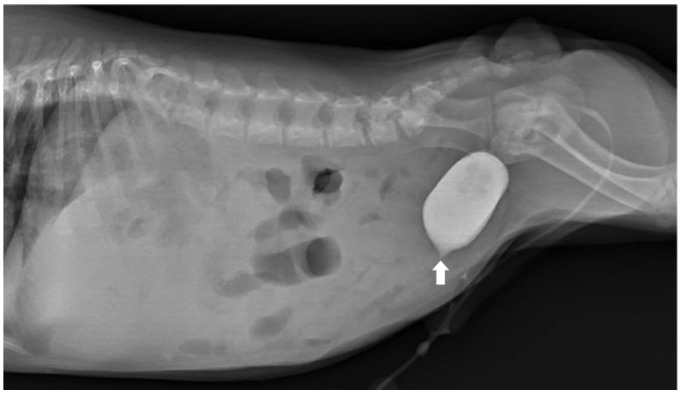
Lateral radiographic view of the abdomen of the dog. Positive contrast cystogram demonstrated a small contrast-filled outpouching at the urinary bladder apex (arrow) without connection to the umbilicus. The dog was diagnosed as having vesicourachal diverticula.

**Figure 3:**
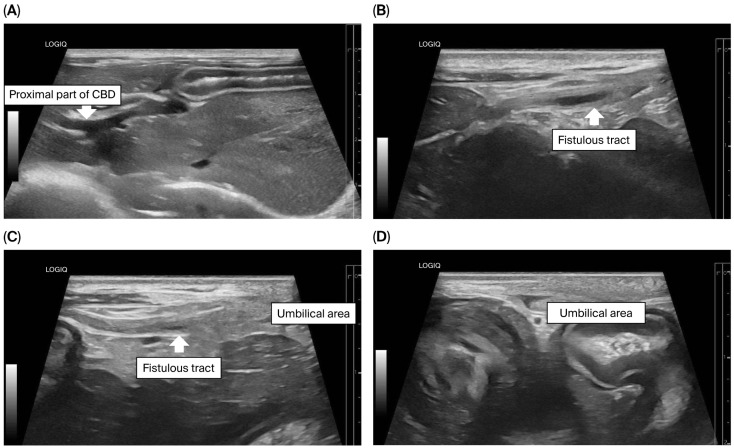
Ultrasonographic findings of the biliary system and umbilical area. (**A**) The proximal part of the common bile duct (CBD) was markedly dilated; (**B**–**D**) A fistulous tract (arrow) extended from the CBD toward the umbilical area.

**Figure 4:**
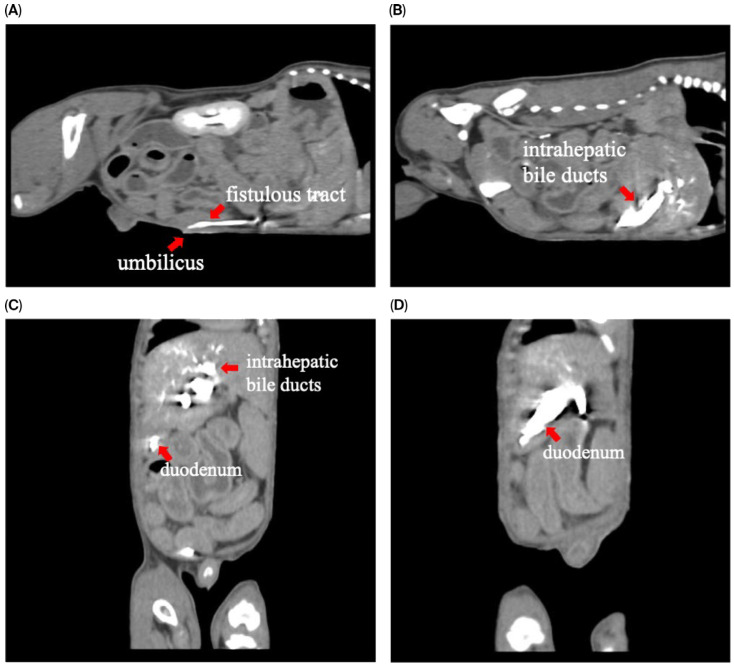
Computed tomography with fistulogram findings demonstrated contrast medium in (**A**) sagittal plane: the fistulous tract extending from the umbilicus; (**B**) sagittal plane: intrahepatic bile ducts; (**C**,**D**) coronal plane: intrahepatic bile ducts and duodenum.

To assess for a persistent urachus, a positive contrast cystogram (KXO-80s, Toshiba, Tokyo, Japan) was performed using iohexal contrast medium (10 mL/kg, diluted to 100 mg I/mL; Omnipaque, GE HealthCare, Bangkok, Thailand). The cystogram demonstrated a contrast-filled structure extending from the apex of the urinary bladder as a convex outpouching of the lumen without evidence of contrast leakage toward the umbilicus (Figure 2). These findings were considered consistent with vesicourachal diverticula. An abdominal ultrasound examination was performed by using a real-time scanner (LOGIQ E9, GE, Fairfield, CT, USA) with a 13 MHz broadband linear transducer. Abdominal ultrasonography revealed a markedly dilated common bile duct (CBD; diameter 0.45 cm) and found an abnormal fistulous tract extending toward the umbilical region, while the gallbladder was not visualized (Figure 3). These findings raised the suspicion of an anomalous extrahepatic biliary tract or an ectopic remnant ductal structure. Therefore, an abdominal computed tomography (CT) scan (Optima 660, GE HealthCare, Bangkok, Thailand) with fistulogram was performed under general anesthesia (induction: propofol [8 mg/kg IV, Troypofol, Troikaa Pharmaceuticals Ltd., Gujarat, India], maintenance: 3% sevoflurane inhalation [Sevo, Singapore Pharmawealth Lifesciences Inc., Laguna, Philippines]). Iohexal contrast medium (2 mL/kg, 300 mg I/mL) was injected via a feeding tube inserted through the umbilicus. The fistulogram demonstrated a tubular tract filled with contrast medium extending from the umbilicus to the CBD and biliary tree, with passage of contrast into the duodenum (Figure 4). The CBD was dilated with contrast medium. In addition, CT imaging revealed the absence of the gallbladder, consistent with agenesis. Together, these findings were compatible with a congenital umbilicobiliary fistula with gall bladder agenesis. All the imaging techniques were performed and interpreted by a veterinary radiologist certified by the Thai Board of Veterinary Surgeons (sub-specialty of veterinary diagnostic imaging).

**Figure 5:**
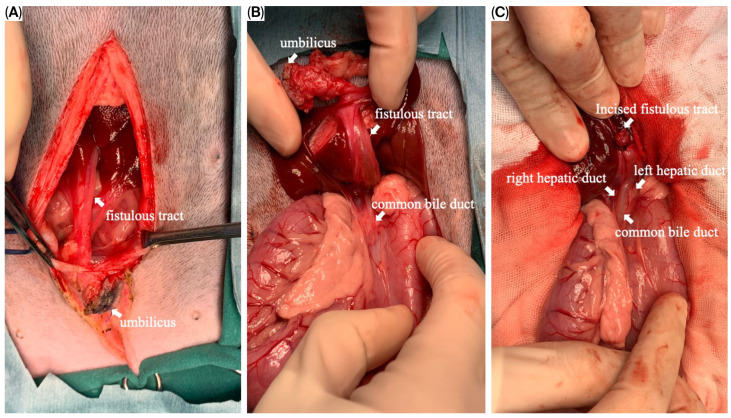
Intraoperative images of the fistulous tract. (**A**) The fistulous tract opened at the umbilical area; (**B**) the fistulous tract, after the dissection from the hepatic fossa, connected between the umbilicus and the common bile duct (CBD); (**C**) the incised fistulous tract terminating at the CBD. The left and right hepatic ducts were demonstrated.

**Figure 6:**
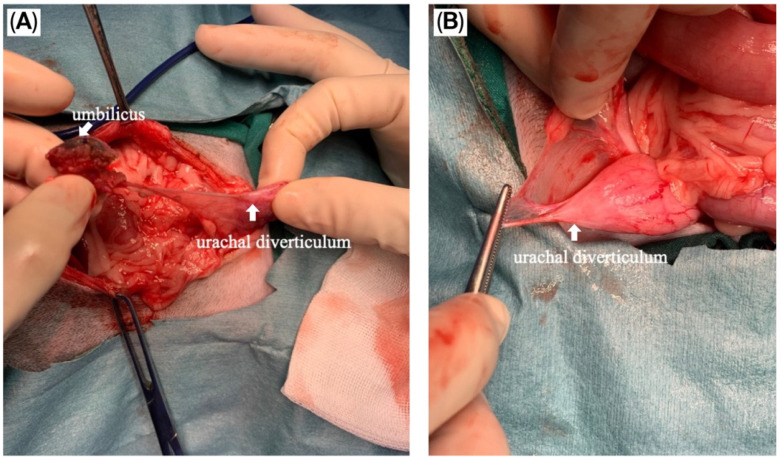
Intraoperative images of the vesicourachal diverticula. (**A**) The thin ligament connecting the urinary bladder and umbilicus was demonstrated; (**B**) Vesicourachal diverticula presented as a small pouch at the apex of the urinary bladder.

The puppy was taken for an exploratory laparotomy when aged 3 months old and weighing 3.8 kg. Generalized anesthesia was induced with slow IV infusion of propofol (4 mg/kg) and maintained with 3.6–5.3% inhalant sevoflurane. Preemptive antibiotic therapy with cefazolin (Cefaben^®^; 20 mg/kg, IV; L.B.S. Laboratory Co. Ltd., Bangkok, Thailand) was given. Morphine sulphate (0.2 mg/kg, IM; M&H Manufacturing Ltd., Samutprakarn, Thailand), fentanyl (1.5 mcg/kg, IV; Siam Bioscience Co., Ltd., Bangkok, Thailand), and fentanyl (10 µg/kg/h CRI) were used for analgesia. The ventral abdominal skin was routinely prepared for surgery. A single simple interrupted suture was placed around the opening of the bile duct at the umbilicus to prevent bile contamination of the surgical area. A standard midline celiotomy was performed by making an incision from the caudal of the umbilicus to the caudal of the abdomen, and cranial of the umbilicus to the xiphoid. A thin-walled fistulous tract that connected the umbilicus area to the CBD was identified (Figure 5A,B). The gallbladder was absent. An incision was made around the umbilicus to free the fistulous tract and the umbilical opening from the abdominal wall. Then, the fistula wall was dissected and separated from the hepatic fossa. The fistulous tract was ligated (polydioxanone 2-0, PDS™II, Ethicon, Johnson & Johnson MedTech (Thailand) Ltd., Bangkok, Thailand) and incised at the opening site near the CBD (Figure 5C). A total of three punch biopsy specimens were obtained from multiple liver lobes. Vesicourachal diverticula and a thin fibrous band connecting the ventro-cranial portion of the bladder to the midline of the abdominal wall at the umbilical area were identified. (Figure 6A). The urinary bladder had a small convex pouch at the apex (Figure 6A,B). The diverticulum and the thin ligament on the urinary bladder were excised. Urinary bladder wall reconstruction was performed using a simple interrupted suture (polydioxanone 4-0). The abdominal wall was closed with the standard procedure.

**Figure 7:**
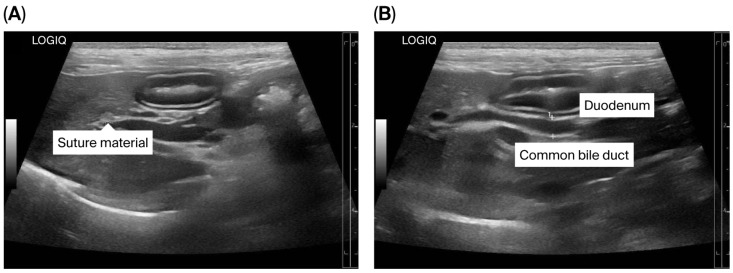
Ultrasonographic findings of the biliary system after surgery. (**A**) Suture material near the common bile duct (CBD) is shown; (**B**) The CBD is mildly dilated.

The puppy’s anesthetic recovery went well. Intravenous fluid therapy using 0.9% NaCl (General Hospital Products Public Co., Ltd., Pathum Thani, Thailand) and antibiotic therapy using cefazolin intravenously (20 mg/kg q12h) were continued for postoperative care. Tramadol (2.5 mg/kg q12h, IV; Tramada-100^®^, L.B.S. Laboratory Ltd., Part., Bangkok, Thailand) was administered for analgesia. Upon recovery, the puppy was bright with a good appetite. The puppy was discharged 72 h after surgery with an uneventful postoperative recovery, and the sutures were removed seven days later. At three months post-operation, ultrasonography demonstrated a smaller size of the CBD (diameter 0.35 cm) (Figure 7A,B). The dog remained clinically healthy without any relevant complications for 19 months post-operation. Unfortunately, the dog died at the age of 1 year 10 months due to aspiration pneumonia of unknown cause.

**Figure 8:**
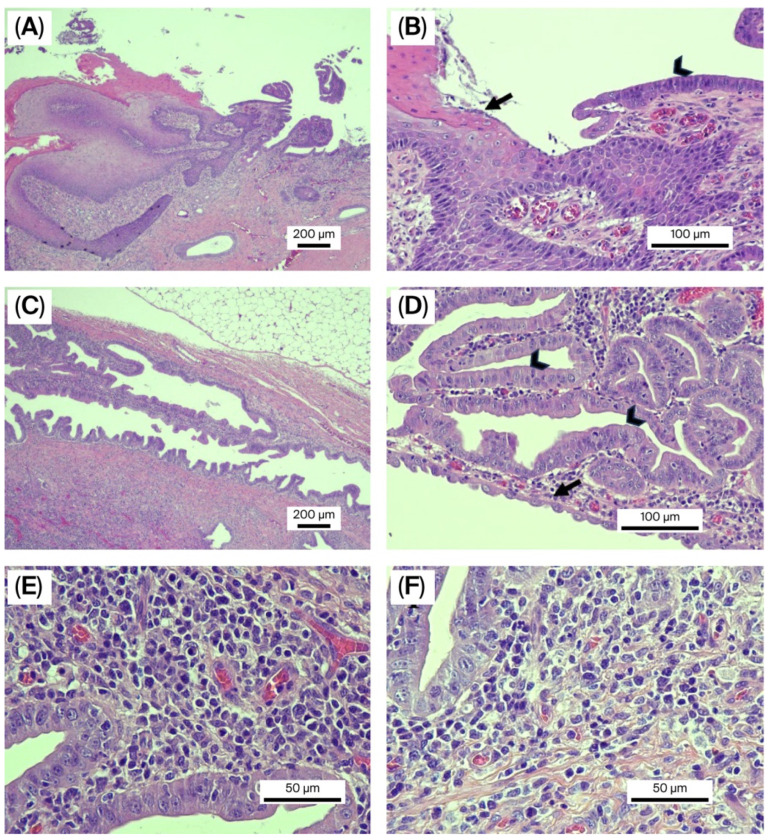
Hematoxylin and eosin staining of the umbilicobiliary fistula. (**A**,**B**) Histopathology of the umbilical skin connected to the bile duct opening at 4× (**A**) and 20× (**B**) magnification. The epidermis was formed by 2–5 layers of squamous epithelial cells producing keratin (arrow). The bile duct was lined by a layer of columnar epithelial cells (arrowhead); (**C**,**D**) Histopathology of the umbilicobiliary fistula at 4× (**C**) and 20× (**D**) magnification. The tissue revealed hyperplastic bile ducts lined by a layer of columnar epithelial cells (arrowhead) that were rearranging into ductal and small papillary patterns. Additionally, cuboidal epithelial cells were also present in some areas (arrow); (**E**,**F**) Histopathology of the umbilicobiliary fistula at 40× magnification shows that the fistulous tract was infiltrated by an abundance of mixed inflammatory cells, primarily lymphocytes, plasma cells, and histiocytes with few neutrophils.

Histopathology of the fistulous tract was analyzed by three board-certified pathology specialists—one American and two Thai. Tissue sections revealed a patent tubular structure consistent with a fistulous tract extending through the dermis and subcutaneous tissue (Figure 8A,B). Most parts of the tract were lined by a single layer of columnar to cuboidal epithelium, which formed small ductal and papillary patterns (Figure 8C,D). This was consistent with the biliary epithelium. The distal lining of the fistulous tract changed from cuboidal/columnar epithelium to squamous epithelium with hyperplasia and metaplasia at the umbilical perimeter. The wall of the tract was composed of a dense proliferation of fibrous connective tissue interspersed with scattered small muscular bundles. A prominent mixed inflammatory cell infiltrate was also presented within the wall, primarily consisting of lymphocytes, plasma cells, and histiocytes, with scattered neutrophils, indicating chronic inflammation surrounding the ductal structure (Figure 8E,F). The histological section of the liver exhibited both vacuolar accumulation in the hepatocytes, i.e., cytoplasmic rarefaction and cellular swelling, especially in centrilobular regions. The cytoplasm contained distinct and clear vacuoles, interpreted as lipids. In addition, the distended portal veins were noted within the larger portal tracts, whereas small or absent portal vein profiles were observed in the smaller portal tracts. There was also a mild increase in the number of bile duct profiles resembling bile duct hyperplasia within the portal tracts. These proliferating ducts appeared as small, closely packed tubules lined by a layer of low-cuboidal epithelial cells. The normal hepatic cord architecture was maintained, with no evidence of collapse or nodular regeneration. The findings suggested a diagnosis of portal vein hypoplasia.

## 3. Discussion

In this case report, we outlined the first case (in both dogs and humans) of three concurrent congenital disorders: umbilicobiliary fistula, gall bladder agenesis, and vesicourachal diverticula. Congenital umbilicobiliary fistula has been reported in three dogs and one human infant [1,2,3,4]. The first dog was a 1-year-old, male, English Bulldog with a normal gallbladder, while the second and third dogs reported were 1-year- and 2-month-old, male, French Bulldog with gallbladder agenesis [1,3,4]. Interestingly, all three previous canine cases involved males, as it did in this case. Therefore, male sex may be a predisposing factor for congenital umbilicobiliary fistula. In addition, the Bulldog breed could be considered as a potential risk factor, as this disease was presented in one English Bulldog and three French Bulldogs. Gallbladder agenesis is a rare condition in dogs [10,11]. Notably, gallbladder agenesis was concurrently identified in three of the four dogs diagnosed with congenital umbilicobiliary fistula, suggesting a potential association between these anomalies. Furthermore, all three cases exhibiting gallbladder agenesis were French Bulldogs, indicating a possible breed predisposition or genetic linkage. These findings warrant further investigation into the embryological development and potential syndromic associations of these congenital biliary anomalies.

To confirm the presence of a congenital umbilicobiliary fistula, several diagnostic modalities were necessary. Although ultrasonography is a non-invasive imaging technique, the definitive diagnosis of an umbilicobiliary fistula cannot be confirmed by this technique. Fistulography is an important tool to confirm the communication of the umbilical opening with the biliary tract. This was the first study to utilize CT imaging to obtain a tentative diagnosis. The imaging techniques assisted in formulating treatment recommendations and surgical planning. Additionally, histopathological evaluation of the fistulous tract revealed that the lining epithelium was most compatible with biliary epithelium. The histopathology and gross anatomical findings served as definitive tools for final diagnosis.

Urachal anomalies are rare congenital disorders of the lower urinary tract, occurring in approximately 0.18% of dogs [12,13]. Four types of urachal anomalies have been identified in dogs and cats, namely patent urachus, urachal cysts, vesicourachal diverticula (extramural and intramural), and urachal sinus [13]. Vesicourachal diverticula are the most frequently diagnosed type of urachal anomaly, with the intramural form being more common, reported in approximately 76.7–88.9% of cases [13,14]. The dog in this case report was identified as having the extramural type of vesicourachal diverticula. Like the present case, most dogs with vesicourachal diverticula have been reported to be asymptomatic [13,14]. In a previous study, 5 out of 50 dogs (10%) showed chronic inflammation of the bladder mucosa [14]. The inflammation of the urinary bladder may be explained by incomplete bladder contraction, which can lead to urinary stasis and accumulation of sediments, especially in dogs with intramural vesicourachal diverticula. Various purebred and mixed dog breeds have been reported to show urachal anomalies. However, French Bulldogs have not previously been reported to have vesicourachal diverticula [13,14].

To the authors’ knowledge, there were no reports of potential complications associated with an uncorrected congenital umbilicobiliary fistula. This could be attributed to the fact that all four dogs (including the present case) underwent surgery within one year of age [1,3,4]. The dog in this case had a mild elevation of serum ALP activity (165 U/L; reference interval [RI] 8–76 U/L), which might originate from the bone isoform of this enzyme due to the patient being a young growing dog [15]. Cholestasis due to inflammation or infection of the fistulous tract lined with biliary epithelium and the biliary system could also lead to an increase in ALP serum activity. Limitations of this present case were that the serum gamma-glutamyl transferase (GGT) activity, another cholestatic marker, was not measured. Neither cytological examination nor bacterial culture of the umbilical discharge was performed. Another limitation of this case report was that testing serum ALP activity was not repeated after surgery.

Liver biopsy was performed in this dog during the surgery. No evidence of ductal plate malformation was found on histological evaluation. Liver histopathological changes in dogs with gallbladder agenesis have been shown to be associated with bile stasis, cholangiohepatitis, and ductal plate malformations [10,11,16]. In the present case, histopathological results demonstrated portal vein hypoplasia. Larger portal tracts had distended portal vein outlines, and this finding may result from a compensatory response to an inadequate number of smaller portal veins to accommodate normal blood flow. The liver histology of a dog with umbilicobiliary fistula and gallbladder agenesis in a previous report demonstrated abnormal portal areas—lacking bile ducts in small portal areas and lacking vessels in larger ones [4]. Biliary reduplication and portal fibrosis were also seen. The portal tracts and hepatic parenchyma showed a distribution of proliferated small arterioles [4].

## 4. Conclusions

We describe the first case of the three concurrent conditions: umbilicobiliary fistula, gallbladder agenesis, and vesicourachal diverticula, in both veterinary and human medicine. Ultrasonography, contrast imaging, and histopathological analysis were performed to obtain a definitive diagnosis. Successful surgical correction of the umbilicobiliary fistula and vesicourachal diverticula was performed when the dog was 3 months old. In addition, umbilicobiliary fistula and gallbladder agenesis may be associated with a vascular malformation of the liver.

## Data Availability

The dataset is available upon request from the authors.

## Abbreviations

The following abbreviations are used in this manuscript:

ALP	Alkaline phosphatase
ALT	Alanine aminotransferase
GGT	Gamma-glutamyl transferase
CBD	Common bile duct
CT	Computed tomography
